# Novel regulation of Ras proteins by direct tyrosine phosphorylation and dephosphorylation

**DOI:** 10.1007/s10555-020-09918-2

**Published:** 2020-09-16

**Authors:** László Buday, Virág Vas

**Affiliations:** 1 Institute of Enzymology, Research Centre for Natural Sciences, Budapest, 1117 Hungary; 2 Department of Medical Chemistry, Semmelweis University Medical School, Budapest, 1094 Hungary

**Keywords:** Ras, Ras signaling, SOS, Tyrosine phosphorylation, SHP2, Cancer therapy

## Abstract

Somatic mutations in the *RAS* genes are frequent in human tumors, especially in pancreatic, colorectal, and non-small-cell lung cancers. Such mutations generally decrease the ability of Ras to hydrolyze GTP, maintaining the protein in a constitutively active GTP-bound form that drives uncontrolled cell proliferation. Efforts to develop drugs that target Ras oncoproteins have been unsuccessful. Recent emerging data suggest that Ras regulation is more complex than the scientific community has believed for decades. In this review, we summarize advances in the “textbook” view of Ras activation. We also discuss a novel type of Ras regulation that involves direct phosphorylation and dephosphorylation of Ras tyrosine residues. The discovery that pharmacological inhibition of the tyrosine phosphoprotein phosphatase SHP2 maintains mutant Ras in an inactive state suggests that SHP2 could be a novel drug target for the treatment of Ras-driven human cancers.

## Introduction

The products of the *RAS* family of proto-oncogenes are low-molecular-weight guanine nucleotide-binding proteins that mediate cell growth, survival, and differentiation *via* interactions with a variety of effector proteins [1, 2]. Ras proteins are enzymes capable of hydrolyzing bound GTP to GDP and inorganic phosphate. Cycling between GDP-bound inactive and GTP-bound active forms is facilitated by guanine nucleotide exchange factors (GEF) and GTPase-activating proteins (GAPs) *via* a mechanism that is common in the Ras superfamily [3, 4]. The three human *RAS* genes encode four highly similar proteins: H-Ras, N-Ras, and K-Ras4A and K-Ras4B. The expression of two protein products from the mammalian *K-RAS* gene results from the use of alternative fourth exons.

Single-base substitutions in codons 12, 13, or 61 of *RAS* are among the most frequent oncogenic mutations in human cancers [5]. These mutations activate Ras by eliminating its GTP hydrolysis activity. Despite the high degree of similarity between the isoforms, K-Ras is the most frequently mutated; indeed, K-Ras mutations have been identified in 22% of all tumors investigated (compared with 8% for N-Ras and 3% for H-Ras) [6, 7].

### Conventional regulation of Ras activation

The first conceptualization of Ras activation was established in the early 1990s. According to this early model, growth factors, e.g., epidermal growth factor (EGF), induce a rapid dimerization and autophosphorylation of their receptors at the plasma membrane. Phosphotyrosine residues in the non-catalytic region of the receptors bind a variety of signaling molecules possessing SH2 or PTB domains, including the Grb2 adaptor protein. Grb2 is a small ubiquitously expressed and highly conserved protein with a central SH2 domain flanked by two SH3 domains. Binding of Grb2 to activated receptors *via* its SH2 domain recruits the SOS-Grb2 complex from the cytosol, placing SOS in proximity to the plasma membrane where it can stimulate the exchange of GDP for GTP on membrane-bound Ras. These early studies suggested that SOS translocation to the plasma membrane was sufficient for Ras activation [8–10].

**Fig. 1:**
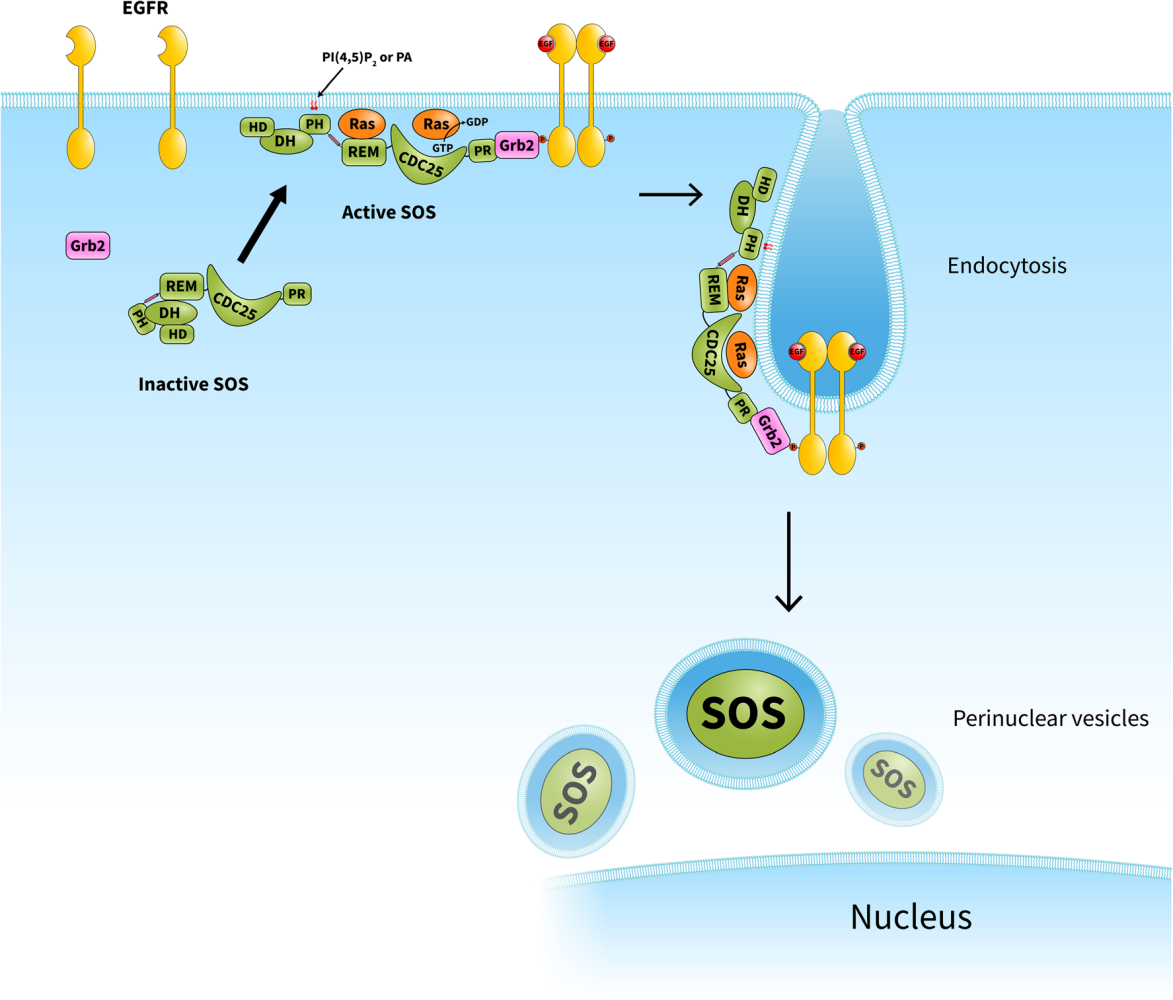
Model for SOS-dependent Ras activation upon growth factor stimulation. In quiescent cells, SOS is maintained in an inactive conformation by the autoinhibitory function of its PH-DH domains. In response to growth factor treatment, SOS is initially recruited to the membrane *via* at least two independent sites: a proline-rich Grb2-binding site and its lipid-binding PH domain. Contact between the PH domain and phospholipids induces conformational changes that allow allosteric Ras binding, which is followed by processive substrate Ras activation. SOS remains bound at the plasma membrane for minutes and is then subjected to endocytosis. As SOS ultimately seems to appear in perinuclear vesicles, it is likely that SOS translocation to the membrane in response to growth factor stimulation is a one-way process. CDC25, CDC25 homology domain; DH, Dbl homology domain; EGFR, epidermal growth factor receptor; HD, histone-like domain; PH, Pleckstrin homology domain; PA, phosphatidic acid; PR, proline-rich domain; REM, Ras exchanger motif

However, the importance of Grb2-mediated membrane recruitment of SOS was challenged by several critical questions emerging from subsequent studies. For example, a transforming mutant of SOS1 that was unable to bind Grb2 seemed to mediate normal downstream signaling [11]. Another study suggested that Grb2 negatively modulates SOS activity under basal conditions [12]. In addition, SOS constructs lacking the Grb2-binding proline-rich regions were found to be successfully recruited to Ras-enriched membranes [13]. How can one explain SOS activation at the plasma membrane if Grb2’s activity is limited at the membrane? The solution may come from the multidomain structure of SOS, which affords it multiple functions, i.e., catalysis, membrane binding (protein-protein and protein-lipid interactions), allosteric regulation, and autoinhibition. SOS possesses a histone-fold domain, Dbl homology (DH) and Pleckstrin homology (PH) domains, a Ras exchanger motif (REM), the catalytic cell division cycle 25 (Cdc25) domain, and a proline-rich C-terminal region that binds Grb2 [10]. Two of the abovementioned domains exert autoinhibitory effects on SOS. The proline-rich region of SOS binds the SH3 domains of Grb2, facilitating SOS recruitment to the plasma membrane. On the other hand, this interaction overcomes negative regulation of SOS by its own C-terminus. Similarly, contact between the SOS PH domain and negatively charged phospholipids also induces conformational changes that allow binding of allosteric Ras to the REM domain, which results in processive catalysis of nucleotide exchange on the substrate Ras in the Cdc25 domain [14–16] (Fig. 1).

Biologically active Ras is strictly localized to membranes determined by specific lipid modifications [17]; therefore, it is not surprising that SOS, upon activation in tyrosine signaling pathway, also binds to membranes containing allosteric and substrate Ras. Furthermore, it has also been shown that constitutive membrane localization of SOS activates Ras [18]. A recent series of elegant experiments from the laboratory of Jay T. Groves shed new light on the dynamics of SOS activation. *Via* single-molecule assays, they identified distinct activation states of SOS, reflecting different stable configurations of the protein complex itself [19]. More interestingly, they recognized a subgroup of SOS molecules in which Ras GTP bound to the SOS allosteric site had a smaller activating effect compared with that of bound Ras-GDP [19]. It seems well-established that SOS translocation to the plasma membrane requires a number of protein-lipid and protein-protein interactions, as described above; however, it has long remained unclear how SOS activity is downregulated in tyrosine kinase signaling. *Via* expression of EGFP-tagged SOS in B cells, they revealed that activated and membrane-translocated SOS remains in the membrane for more than 10 min. By half an hour after stimulation, most SOS protein seemed to appear in perinuclear vesicular structures, suggesting that SOS is incapable of dissociating from the membrane, instead being subjected to endocytosis [13] (Fig. 1).

### Ras inhibition by direct tyrosine phosphorylation

**Fig. 2:**
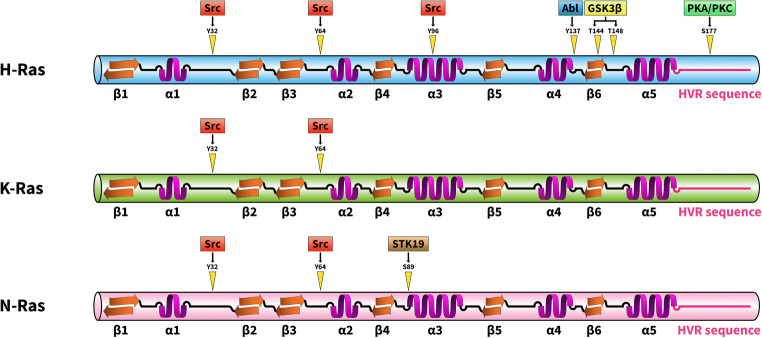
Phosphorylation site of Ras proteins. Schematic representation of the phosphorylation sites discussed in this review. GSK3β, glycogen synthase kinase 3β; PKA, protein kinase A; PKC, protein kinase C; STK19, serine/threonine-protein kinase 19

In the first report on Ras phosphorylation in 1989 [20], the authors reported that purified H-Ras was phosphorylated *in vitro* by insulin receptor in the presence of poly(L-lysine). Interestingly, poly(L-lysine) was not necessary for K-Ras phosphorylation in the same assay system, perhaps due to the unique, extremely basic hypervariable region of K-Ras, which might effectively substitute for the poly(L-lysine). Abl tyrosine kinase was also reported to phosphorylate H-Ras on tyrosine residue 137 [21]. Tyrosine phosphorylation of H-Ras by Abl allosterically enhanced the binding of H-Ras to its effector protein Raf. Serine/threonine phosphorylation of Ras proteins may also play important roles in their regulation. For example, H-Ras phosphorylation by GSK3β on threonine residues 144 and 148 results in polyubiquitination and proteasome-mediated degradation [22]. Protein kinase C–dependent K-Ras4B phosphorylation was also found on Ser181, which is located in the C-terminal hypervariable polybasic region. Phosphorylation of K-Ras S181 induces rapid translocation of the protein from the plasma membrane to internal membranes, including the endoplasmic reticulum and the mitochondrial membrane. Since phosphorylated K-Ras interacts with Bcl-xL at the outer mitochondrial membrane, K-Ras was implicated in cell death regulation [23, 24]. Recently, a poorly characterized serine/threonine kinase, STK19, was identified as a novel activator of N-Ras [25]. STK19 phosphorylates N-Ras on the evolutionarily conserved Ser89 in the protein’s α3-helix. Ser89 phosphorylation facilitates interactions between N-Ras and its downstream effectors, e.g., B-Raf and PI3Kα, resulting in increased activation of the MAP kinase cascade and the PI3K pathway. The potential therapeutic effect of a STK19 inhibitor was successfully tested in a melanoma model. It was shown that the selective STK19 inhibitor ZT-12-037 (1a) markedly inhibited N-Ras–driven melanoma development and growth both *in vitro* and in animals [25] (Fig. 2).

The cooperation between Src and Ras in cell growth regulation and tumorigenesis has been known for years. For example, both Ras and Src are downstream members of epidermal growth factor (EGF) signaling [8, 9, 26]. Viral Src can phosphorylate the adaptor protein Shc, which then recruits Grb2/SOS complexes for Ras activation at the plasma membrane [27]. Ras has been shown to be essential for v-Src–stimulated cell transformation of human gallbladder epithelial cells or invasive pancreatic ductal adenocarcinoma [28, 29]. p120RasGAP was identified as an effector of c-Src activation by oncogenic Ras [30]. Furthermore, Src cooperates with mutant Ras in tumourigenesis through the JNK and PI3K pathways [31], although no direct relationship between Src and Ras has been identified in this process. Recent studies from the laboratory of Michel Ohh demonstrated that Src tyrosine kinase directly phosphorylates Ras on tyrosine 32 within the switch I region [32]. Interestingly, Src can bind to and phosphorylate Ras, but only the GTP-bound form; therefore, it is highly likely that Src distinguishes between the different conformations of Ras and can bind only to the activated G protein. Ras tyrosine 32 phosphorylation has two important effects on Ras signaling: phosphorylation inhibits the binding of the effector protein Raf while increases the binding of the GAP protein leading to enhanced GTP hydrolysis [32]. Both changes facilitate a conformational shift in Ras that inactivates the Ras cycle.

Real-time nuclear magnetic resonance experiments have shown that Src kinase phosphorylates not only Tyr32 in K-Ras but also Tyr64 [33]. This activity is particularly interesting since Tyr32 is located in the switch I region (aa 30-38) while Tyr64 is positioned in the switch II sequence of K-Ras (aa 59-76). These regions of Ras undergo conformational changes when GDP is exchanged for GTP. In the GTP-bound state, Tyr35 and Gly60 form hydrogen bonds with the γ-phosphate and lock the switch I and II regions in the active conformations [17]. Both the switch I and II regions are implicated in the binding of effectors and GAP proteins. After GTP hydrolysis, the γ-phosphate is released and both switch regions return to the flexible conformation in the GDP-bound state [17]. Considering the important roles of the switch regions in Ras regulation, it is not surprising that Tyr32 and Tyr64 phosphorylation markedly alters the conformation of the switch I and II regions, negatively influencing every step of the Ras cycle [33]. As such, K-Ras tyrosine phosphorylation significantly reduces the associated GEF and GAP activities and impairs binding of the Raf effector molecule [33].

The crystal structure of nucleotide-free Ras in complex with the catalytic domain of SOS revealed that SOS induces conformational changes in the switch I and II regions of Ras [34]. Mutagenesis of residues at the switch II-SOS interface showed that the primary contact residue is Tyr64, which was buried in a hydrophobic pocket of SOS [35]. Interestingly, the switch I region of Ras is also involved in the interaction with SOS. Mutagenic analysis revealed that Tyr32 and Tyr40 play fundamental roles in this contact. Mutation of these residues resulted in an increase in the intrinsic rate of nucleotide exchange and decreased the binding of Ras to SOS [35]. These findings largely explain why Tyr32 and Tyr64 phosphorylation in Ras, resulting in the incorporation of phosphate groups at the contact sites of Ras and SOS, impairs GEF activity.

### Ras activation by the SHP2 phosphoprotein phosphatase

SH2 domain-containing protein tyrosine phosphatase 2 (SHP2), encoded by *PTPN11*, is associated with a number of malignant conditions, including breast cancer, leukemia, lung cancer, liver cancer, gastric cancer, and other cancer types [36]. Germline-activating mutations in the *PTPN11* gene cause Noonan syndrome, whereas somatic mutations result in LEOPARD syndrome and childhood leukemia [36]. The protein phosphatase contains two SH2 domains, a PTP catalytic domain and a C-terminal tail. The crystal structure of the SHP2 tyrosine phosphatase has revealed how its catalytic activity is regulated by its two SH2 domains. In the absence of a tyrosine-phosphorylated–binding partner, SHP2 is kept in an autoinhibited conformation by intramolecular interactions between the N-terminal SH2 domain and the phosphatase domain. However, in response to growth factor or cytokine stimulation, binding of specific phosphotyrosine proteins to the N-terminal SH2 domain releases the autoinhibition and activates SHP2. Recognition of bisphosphorylated ligands by the tandem SH2 domains is an integral element of the activation. The C-terminal SH2 domain contributes binding energy and defines specificity, but it does not have a direct role in activation [36, 37].

In 2007, *PTPN11* (the SHP2-encoding gene) was identified as the first proto-oncogene that encodes a tyrosine phosphatase [38]. Although phosphatases are generally thought to be negative regulators of signaling pathways involving protein kinases, several lines of evidence suggest that SHP2 promotes growth factor- and cytokine-induced Ras activation [39]; therefore, SHP2 has long been considered as a potential therapeutic target in cancer treatment. It was demonstrated that SHP2 inhibition blocks signaling from receptor tyrosine kinases (e.g., the EGF receptor) to the MEK-ERK pathway [40]. SHP2 inhibition seemed lethal to cells that are driven by activated tyrosine kinases, and it was implicated in intrinsic and acquired resistance to targeted cancer drugs [40]. Other data demonstrated that pharmacological SHP2 inhibition is an effective therapeutic approach for cancer treatment. A selective and orally bioavailable small-molecule SHP2 inhibitor, SHP099, can block the Ras-Erk signaling pathway activated by mutant receptor tyrosine kinases [41]. Interestingly, SHP099 binding to the interface of the N-terminal SH2 domain and the tyrosine phosphatase domain stabilizes SHP2 in the autoinhibited confirmation [41]. SHP2 inhibition alone had very little or no effect on proliferation in non-small-cell lung cancer (NSCLCs) cell lines; however, a combination of SHP2 and MEK inhibitors showed marked synergy in K-RAS-mutant NSCLC cells and in animal models [42, 43]. Finally, studies in which SHP2 was targeted with the small allosteric inhibitor RMC-4550 demonstrated that it can be an effective target in B-Raf mutant cancer cell lines. It was proposed that the mechanism of the inhibitory effect involves disruption of SOS-mediated Ras GTP loading [44].

**Fig. 3:**
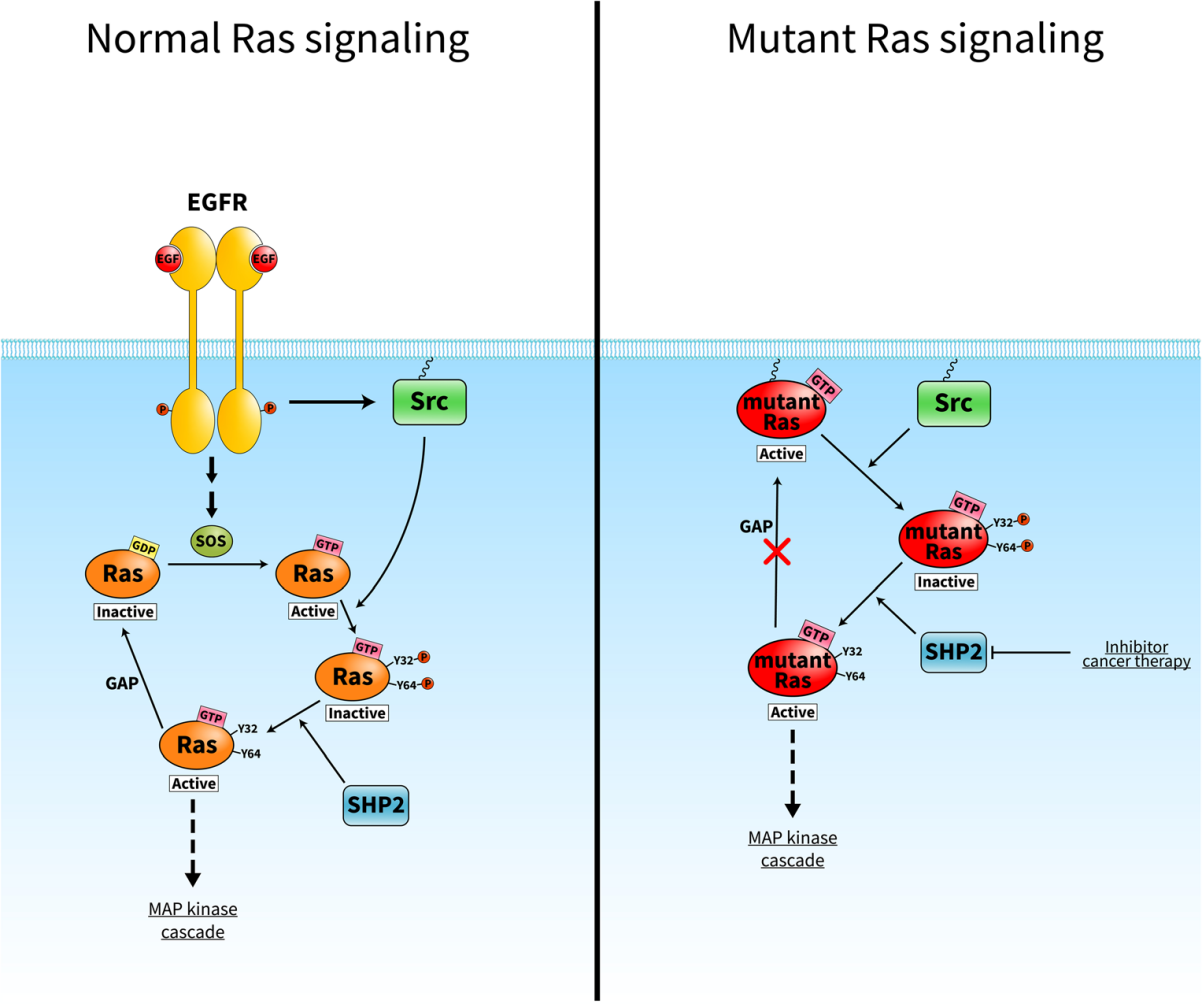
Proposed model of the phosphorylation-dependent Ras GTPase cycle*. Normal Ras signaling*: EGF receptor stimulation leads to simultaneous activation of the SOS exchange factor and Src tyrosine kinase. GTP-bound Ras is a substrate of Src, which phosphorylates Ras on tyrosine 32 and tyrosine 64, resulting in Ras inactivation. Release from this inhibition can be achieved *via* the action of SHP2 phosphatase, which dephosphorylates Ras protein. *Mutant Ras signaling*: oncogenic mutations activate Ras *via* a defect in its ability to hydrolyze GTP, thus locking Ras in a GTP-bound state. Src phosphorylates the constitutively active Ras, thus leading to its inactivation. SHP2 dephosphorylates Ras allowing downstream signaling. Therefore, SHP2 is a novel drug target that might be effective in Ras-driven human cancer

Although several promising SHP2 inhibitors for use in cancer therapy have been described in the last decade, the precise mechanism by which SHP2 promotes Ras activation remains fundamentally unclear. Bunda et al. identified for the first time that SHP2 preferentially binds to and dephosphorylates PTyr32 Ras [45]. They showed that SHP2 dephosphorylates wild-type and mutant Ras, and the activities of SHP2 and Ras were elevated in mouse and human glioblastoma multiforme cell lines. In addition, SHP2 inhibition facilitated phosphorylation of wild-type and oncogenic Ras, resulting in inactivation of Ras and its downstream signaling [45]. The above findings suggest the existence of another layer of Ras regulation that might fine-tune the classical GEF/GAP-mediated Ras cycle. In response to, for example, EGF stimulation, Src tyrosine kinase is activated at the plasma membrane where it preferentially phosphorylates GTP-bound Ras in which the Tyr32 hydroxyl-group is rotated in the optimal position for phosphorylation [32]. Tyrosine phosphorylation negatively regulates Ras, even in its GTP-bound state, and blocks the Ras cycle. Release from this inhibition can be achieved *via* the action of SHP2 phosphatase, which dephosphorylates Ras protein (Fig. 3). What is the rationale behind this regulation in terms of cancer treatment? Ras mutations lead to decreased intrinsic Ras GTPase activity and/or impaired binding of GAP protein to Ras, both of which lead to sustained and elevated Ras activation. Mutant GTP-bound Ras acts as a driver that contributes to the pathogenesis of several cancer types. For many decades, Ras was thought to be an “undruggable” cancer target, since no drug against Ras had been approved by authorities [46]. The discovery that SHP2 can dephosphorylate even mutant GTP-bound Ras in tumor cells and release Ras from its inactive state establishes SHP2 as a novel drug target that could be effective in Ras-driven human cancer.
